# Robotic distal pancreatectomy using two-surgeon technique (TAKUMI-4): a technical note and initial outcomes

**DOI:** 10.1007/s00423-025-03751-3

**Published:** 2025-06-02

**Authors:** Kosei Takagi, Tomokazu Fuji, Kazuya Yasui, Toshiyoshi Fujiwara

**Affiliations:** Department of Gastroenterological Surgery, Okayama University Graduate School of Medicine, Dentistry, and Pharmaceutical Sciences, 2-5-1 Shikata-cho, Kita-ku, 700-8558 Okayama Japan

**Keywords:** Distal pancreatectomy, Robotic surgery: minimally invasive surgery, Training, Outcomes

## Abstract

**Purpose:**

With the increasing use of minimally invasive distal pancreatectomy, the use of robotic distal pancreatectomy (RDP) is also increasing worldwide. Standardized surgical protocols are essential for safe implementation of RDP. In this study, we present our surgical protocol and initial outcomes of RDP using “two-surgeon technique”.

**Methods:**

Our standard RDP protocol included a two-surgeon technique for cooperation, rationality, and education. Short-term outcomes of RDP were also investigated. This retrospective study included 77 consecutive patients who underwent RDP at our institution between April 2021 and January 2025.

**Results:**

The median operative time, estimated blood loss, and postoperative hospital stay were 214 min (interquartile range [IQR], 176–253), 10 mL (IQR, 0–50), and 9 days (IQR, 8–10), respectively. A textbook outcome was achieved in 84.4% of patients. Moreover, superior outcomes of RDP (*n* = 77) compared with those of laparoscopic distal pancreatectomy (*n* = 62) were confirmed in this study.

**Conclusion:**

Using the two-surgeon technique, we successfully standardized and introduced the RDP program. The two-surgeon technique can contribute to the safe introduction of RDP and expansion of the program.

## Introduction

With advancements in surgical techniques and instruments, minimally invasive distal pancreatectomy (MIDP) has become internationally popular and is supported by growing evidence [1]. Moreover, several studies have suggested the safety and oncological benefits of laparoscopic DP (LDP) and robotic DP (RDP) compared with conventional open DP [2]. With the increasing use of MIDP, the number of robotic approaches for distal pancreatectomy has also increased [3]. Robot-specific formal training and the establishment of standardized surgical protocols are required to safely implement RDP.

Surgical approaches to robotic surgery can be divided into purely robotic and robot-assisted techniques [3]. In the purely robotic technique, the procedure is performed using only robotic instruments with no laparoscopic energy devices. Robot-assisted techniques include pancreatic dissection using both robotic and laparoscopic instruments. Considering the limited robotic instruments and the complexity of pancreatic surgery, the robot-assisted technique may be suitable for RDP, especially in the initial phase. In the robot-assisted technique, the “two-surgeon technique” focuses more on cooperation between the console and the assistant surgeon (patient-side surgeon) during the procedure. Although the usefulness of the two-surgeon technique has been demonstrated in robotic hepatectomy [4], few studies have described the surgical techniques of RDP focusing on the two-surgeon technique.

The aim of this study was to demonstrate our surgical protocol and initial outcomes of RDP using the two-surgeon technique and compare with those of LDP. Our study falls under Training program in Okayama University for minimally invasive surgery (TAKUMI-4).

## Materials and methods

### Training model

The structured training model for console surgeons includes basic robotics, simulators, biotissue, video training, and clinical experience in robotic surgery [5]. A step-by-step robotic curriculum that includes intraoperative coaching and evaluation offers a safe integration option for complex robotic skills. Because the role of the patient-side surgeon is important in robotic surgery, the patient-side surgeon needs to understand the robotic system and gain clinical experience in robotic surgery.

### Two-surgeon technique

The two-surgeon technique is a method in which both the console and patient-side surgeons understand their roles in various situations, cooperate with each other, and consider educational aspects to perform rational surgery. The two-surgeon technique allows the pursuit of rationality and optimization of procedures, resulting in shortened operative time.

**Fig. 1:**
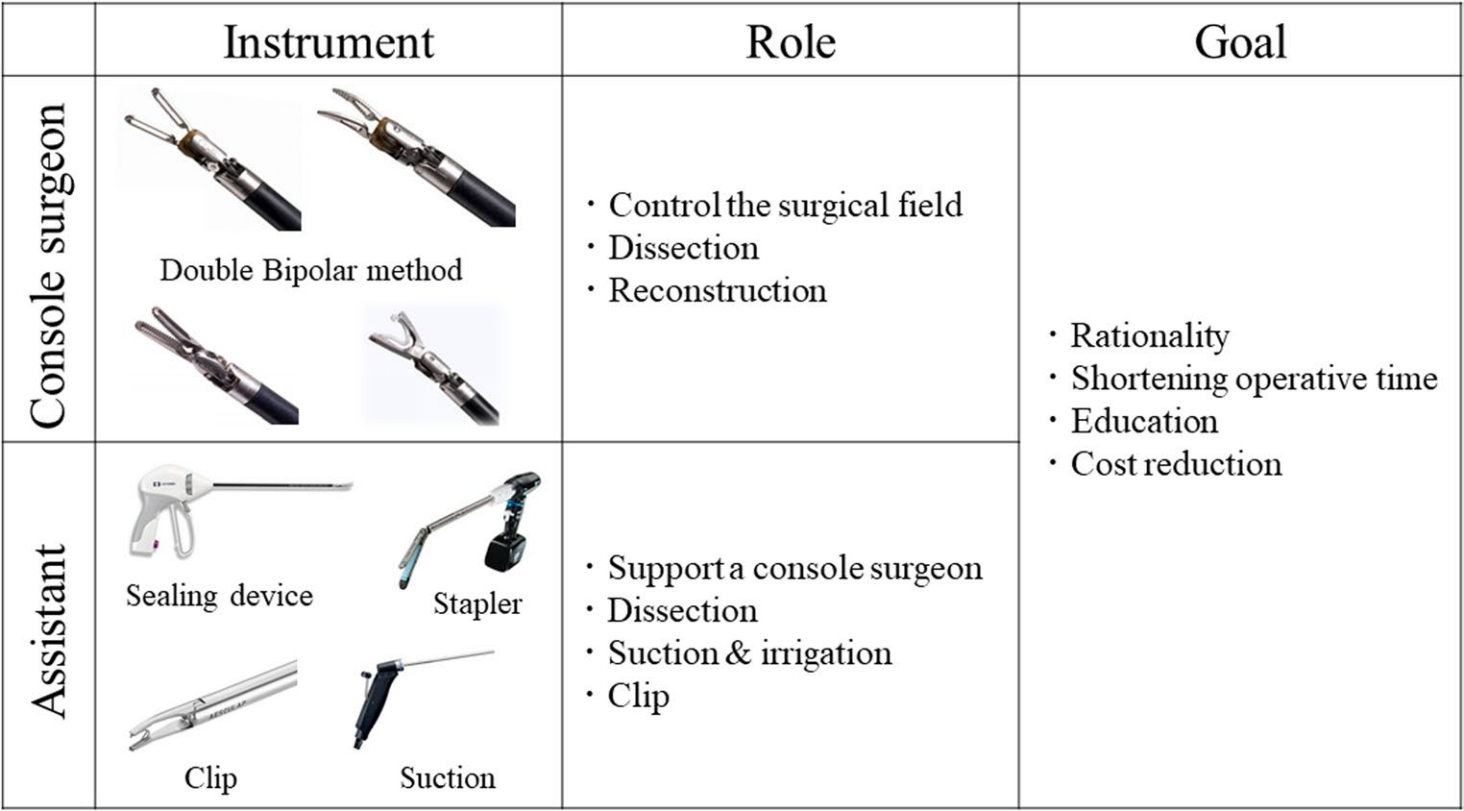
The distribution of instruments and roles in two-surgeon technique for robotic distal pancreatectomy

The surgeons’ roles are briefly summarized in Fig. 1. A console surgeon controls the surgical field and performs dissection and reconstruction. A patient-side surgeon can support a console surgeon using laparoscopic devices such as suction and clips. Appropriate surgical devices should be selected by the console or patient-side surgeon. Minimizing the changes in the robotic arms may stabilize the surgical field and reduce operative time.

### Surgical protocol for RDP

**Fig. 2:**
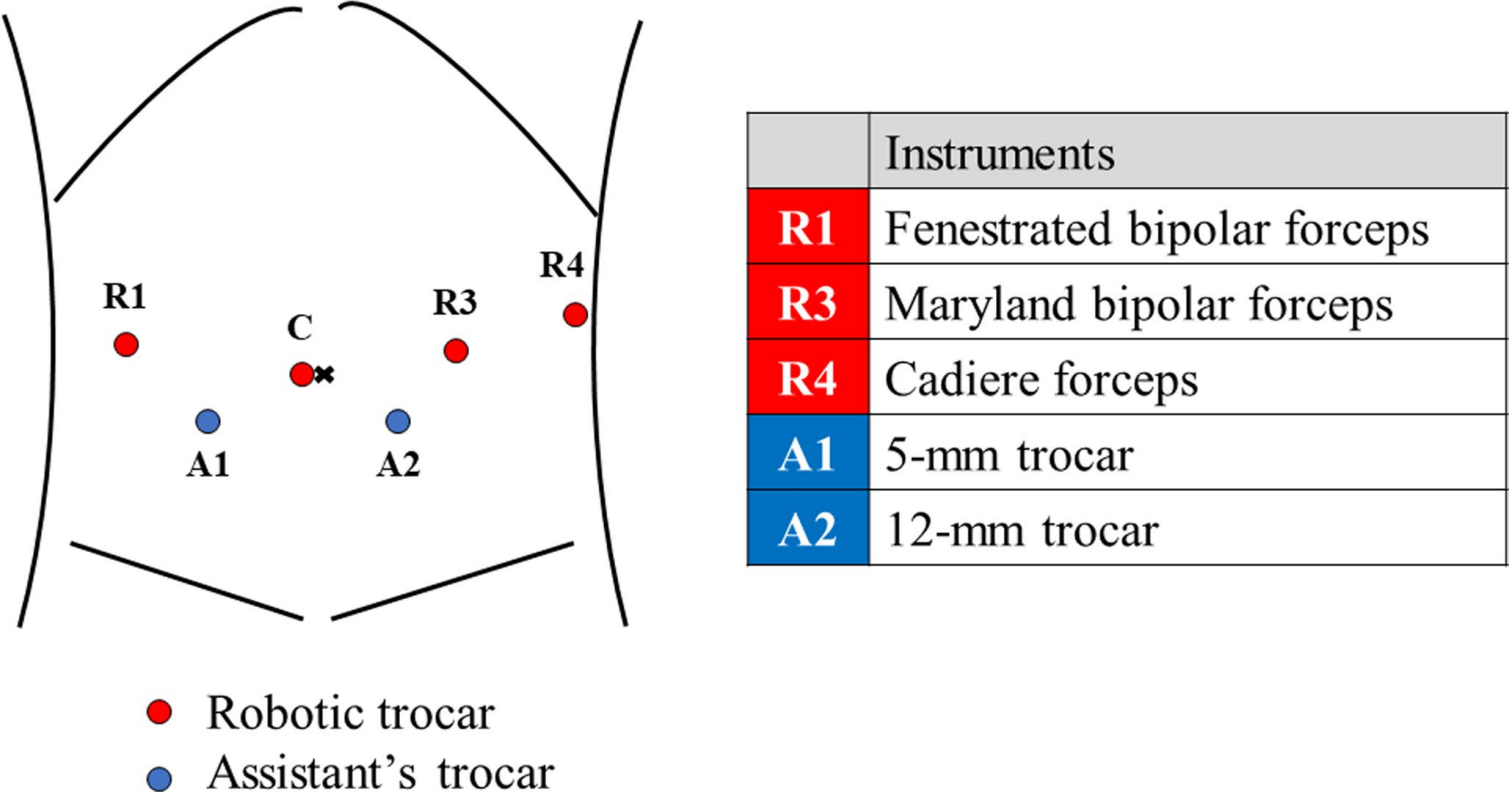
Trocar placement in robotic distal pancreatectomy

RDP was performed using a two-surgeon technique. Patients were placed in the seven-degree reverse Trendelenburg position and seven-degree tilt right using the TS7000 dV operating table, with a patient-side surgeon between the legs. The trocar placement procedure is illustrated in Fig. 2. The standard RDP technique can be divided into four steps. Initially, the gastrocolic ligament was divided to approach the pancreas (step 1). Otherwise, the gastrohepatic ligament approach can be applied to dissect the pancreas. Next, the pancreas was dissected (step 2), followed by the division of the pancreas and splenic vessels (step 3). A surgical strategy for approaching the splenic artery has been reported previously [6]. Finally, the pancreas was mobilized using a medial approach. Radical antegrade modular pancreatosplenectomy technique was used for retroperitoneal dissection in patients with pancreatic cancer. The spleen-preserving technique with a splenic vessel-preserving approach (Kimura technique) or a splenic vessel-sacrificing approach (Warshaw technique) can be considered in cases of benign tumors. All RDPs in the study period were performed using the two-surgeon technique and proctored by a single surgeon (KT) who received comprehensive robotic training in the Netherlands [5].

### Perioperative management

The principle of enhanced recovery after surgery protocols was applied as previously reported [7]. The preoperative factors included mobilization, counseling, oral carbohydrate loading, and no preanesthetic medication. Patients were managed by total intravenous anesthesia. The postoperative factors included early scheduled mobilization, early oral intake, and early removal of the urinary catheter and strict glycemic control by diabetologists. The abdominal drain was placed routinely and removed when no clinically relevant postoperative pancreatic fistula (POPF) was detected.

### Data collection and statistical analysis

This study included 77 consecutive patients who underwent RDP at our hospital between April 2021 and January 2025. Regarding our MIDP strategy, we introduced the LDP in June 2012 and the RDP in April 2021. After October 2021, all MIDP will be performed using robotic approach.

Using a prospectively collected database, the following data were extracted: age, sex, body mass index, American Society of Anesthesiologists physical status, hypertension, diabetes, neoadjuvant chemotherapy, primary disease, operative time, estimated blood loss, conversion to open surgery, hand-assisted surgery, mortality, major complication (Clavien-Dindo grade ≥ 3) [8], POPF (≥ grade B) [9], postpancreatectomy hemorrhage, postoperative hospital stay, and readmission. All complications that developed within one month after surgery were collected. The absence of mortality, major complications, POPF, postpancreatectomy hemorrhage, and readmission were regarded as textbook outcome [10].

Values are presented as proportions for categorical data and medians (interquartile ranges [IQR]) for continuous variables. The outcomes of the RDP were investigated and compared with those of the LDP (*n* = 62) between June 2012 and January 2025. All statistical analyses were performed using JMP software version 11 (SAS Institute, Cary, NC, USA).

### Ethical approval

This study was approved by the Ethics Committee of Okayama University Hospital and was performed in accordance with the tenets of the Declaration of Helsinki.

## Results

**Fig. 3:**
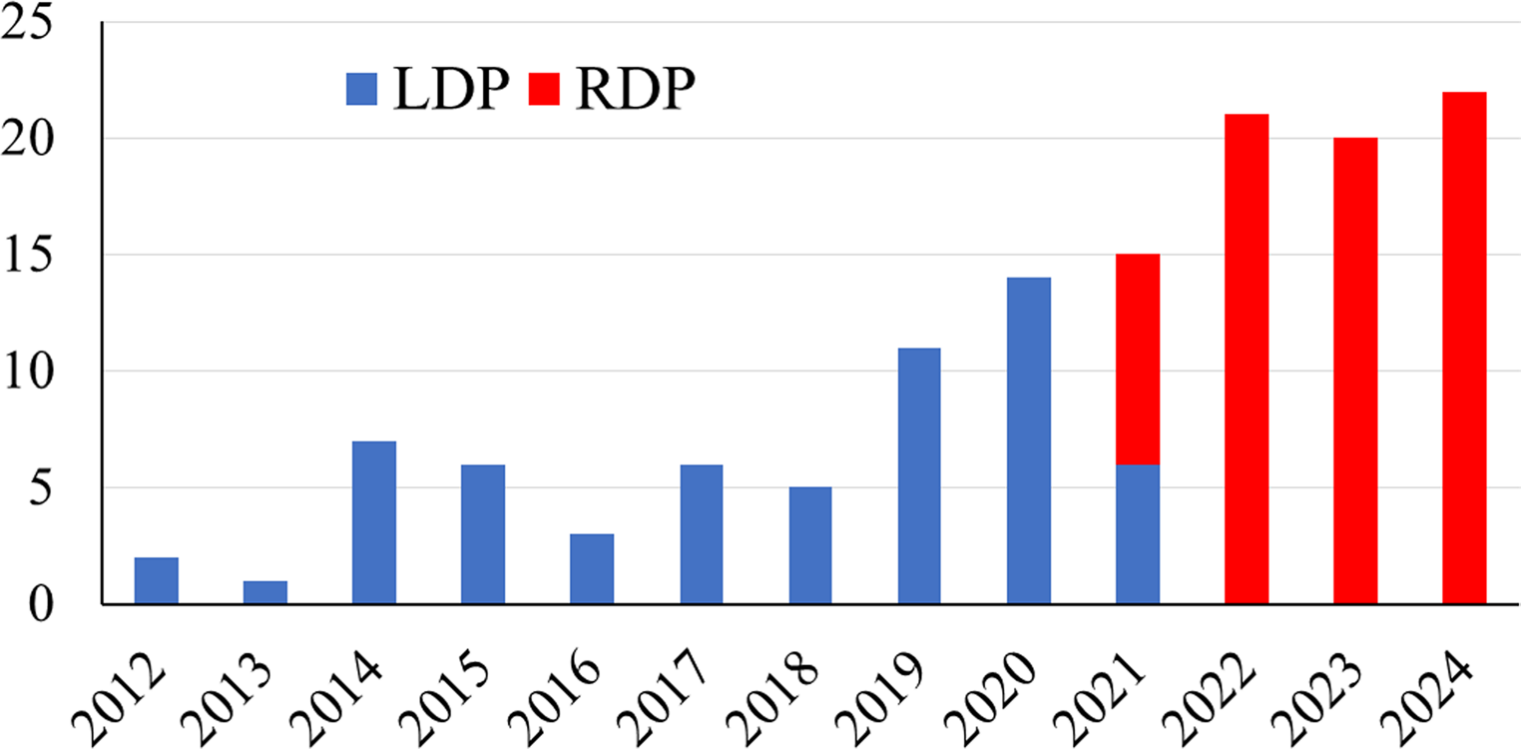
The annual number of minimally invasive distal pancreatectomy at our institution. Laparoscopic distal pancreatectomy was introduced in June 2012, and robotic distal pancreatectomy in April 2021. After October 2021, all procedures were performed using robotic approach

The annual number of MIDP, including 77 RDP and 62 LDP, is shown in Fig. 3. Patient characteristics and outcomes of RDP are summarized in Table 1. There were 31 men and 46 women, with a median age of 70 years (IQR, 56–76 years). Half of the primary diseases were malignant, including pancreatic cancer (*n* = 35) and metastatic pancreatic tumors (*n* = 3). The median operative time and estimated blood loss were 214 min (IQR, 176–253 min) and 10 mL (IQR, 0–50 mL), respectively. The spleen-preserving technique was performed in 37 cases: 13 using the Kimura technique and 24 using the Warshaw technique. The incidence rates of mortality, major complications, and POPF (≥ grade B) were 0%, 6.5%, and 10.4%, respectively. The median postoperative hospital stay was 9 days (IQR, 8–10 days). A textbook outcome was achieved in 84.4% of patients.

**Table 1:** Initial outcomes of robotic distal pancreatectomy comparing to laparoscopic distal pancreatectomy

	RDP (*n* = 77)	LDP (*n* = 62)	*P* value
Age, years	70 (55–76)	66 (48–72)	0.025
Sex			
Men	31 (40.3)	25 (40.3)	0.99
Women	46 (59.7)	37 (59.7)	
BMI, kg/m^2^	23.0 (20.3–26.4)	22.6 (19.0–25.8)	0.31
ASA			
1–2	62 (80.5)	54 (87.1)	0.30
3–4	15 (19.5)	8 (12.9)	
Hypertension	36 (46.8)	27 (43.6)	0.71
Diabetes	31 (40.3)	13 (21.0)	0.01
Neoadjuvant chemotherapy	27 (35.1)	9 (14.5)	0.005
Primary diseases			
Malignancy	38 (49.4)	13 (21.0)	< 0.001
Benign	39 (50.6)	49 (79.0)	
Operative time, min	214 (176–253)	334 (252–405)	< 0.001
Blood loss, mL	10 (0–50)	110 (18–219)	< 0.001
Conversion	0 (0)	2 (3.2)	0.07
Hand-assisted	0 (0)	8 (14.8)	-
SPDP	37 (48.1)	7 (11.3)	< 0.001
Kimura	13 (35.1)	7 (100)	
Warshaw	24 (64.9)	0 (0)	
Mortality	0 (0)	0 (0)	-
Major complications (CDc ≥ 3)	5 (6.5)	20 (32.3)	< 0.001
POPF (≥ grade B)	8 (10.4)	14 (22.6)	0.05
PPH	0 (0)	3 (4.8)	0.03
Hospital stay, day	9 (8–10)	13 (10–18)	< 0.001
Readmission	9 (11.7)	4 (6.5)	0.28
Textbook outcome	65 (84.4)	41 (66.1)	0.01

Values are reported as n (%), or median (interquartile range)

*BMI* body mass index; *ASA* American Society of Anesthesiologists; *SPDP* spleen-preserving distal pancreatectomy; *CDc* Clavien–Dindo classification; *POPF* postoperative pancreatic fistula; *PPH* postpancreatectomy hemorrhage

The outcomes of the RDP and LDP groups are shown in Table 1. Although LDP was performed for more benign tumors, better operative outcomes were confirmed with RDP, with a significantly shorter operative time (RDP vs. LDP; 214 vs. 334 min, *P* < 0.001) and less blood loss (10 vs. 110 mL, *P* < 0.001). Moreover, significantly lower incidences of postoperative complications were found in the RDP group, leading to shorter postoperative hospital stays (9 vs. 13 days, *P* < 0.001) and better textbook outcomes (84.4 vs. 66.1%, *P* = 0.006).

## Discussion

This study presents the concept of the two-surgeon technique and our surgical protocol for RDP. Moreover, the initial outcomes of RDP were compared with those of LDP. Using the two-surgeon technique, we successfully introduced an RDP program with outcomes superior to those of LDP.

The two-surgeon technique has several advantages. Pancreatic dissection performed by a console and patient-side surgeon allows less exchange of robotic instruments, leading to a reduced operative time. The limited use of robotic instruments can reduce surgical costs. Moreover, the educational benefits of the two-surgeon technique should be emphasized. An experienced console surgeon can educate inexperienced patient-side surgeons and vice versa. The roles of the console and the patient-side surgeons depend on their surgical experiences and skills. After gaining adequate individual and institutional experience in robotic surgery, surgeons can select pure robotic or robot-assisted techniques based on their preferences.

Our structured training model for robotic surgery was developed on the basis of a multicenter nationwide training program for robotic pancreatectomy in the Netherlands (LAELAPS-3) [5]. After completing the training program, we standardized the surgical protocols and introduced RDP using the two-surgeon technique in Japan. The experiences of LAELAPS-3 should contribute our better outcomes of RDP compared with benchmark cutoffs of RDP, including operation time ≤ 300 min, conversion rate ≤ 3%, clinically relevant POPF ≤ 32%, and 3 months major complication rate ≤ 26.7% [11]. Moreover, the textbook outcome achievement rate in our study was higher than that of international expert centers [12]. In this study, the Warshaw technique was more frequently selected in RDP, considering the potential benefits of splenic preservation. The precise intraoperative hemodynamics evaluation of the spleen using indocyanine green fluorescence imaging contributed to higher chances of the spleen-preserving technique during RDP.

A recent systematic review comparing RDP to LDP showed that RDP was associated with lower conversion rates and shorter hospital stay, but had similar incidences of postoperative complications, except for 30-day mortality [13]. Although we found superior outcomes of RDP compared with LDP in this study, the differences in historical background and surgeons’ experiences may have resulted in these findings. As this was a single-center retrospective investigation with a relatively small sample size, our results may have been influenced by potential selection bias and confounding factors.

There were several limitations in this study. This study was conducted in a single center with a relatively small sample size, potentially leading to selection bias. Moreover, the backgrounds of surgical indications and surgeons differed between RDP and LDP. Regarding the national insurance system in Japan, LDP was officially allowed for benign tumors in 2012 and for malignant tumors in 2016, followed by an allowance of RDP for benign and malignant tumors in 2020. Regarding surgeons’ backgrounds, all RDPs were performed by a single surgeon (KT) who had adequate experience of robotic surgery. Therefore, the learning curve of RDP would be limited in this study. In contrast, LDP was mainly performed by another surgeon who had inadequate experience of LDP prior to starting the program. Therefore, outcomes of LDP may be influenced by the learning curve. These different backgrounds between RDP and LDP may affect different postoperative outcomes. Regarding surgical technique, all RDPs were performed using the two-surgeon technique. Therefore, comparing the two-surgeon technique with other robotic approaches was not possible.

## Conclusion

This study demonstrates our surgical protocol and initial outcomes of RDP using a two-surgeon technique. The technique can contribute to the safe introduction of RDP and expansion of the program.

## Data Availability

No datasets were generated or analysed during the current study.
